# Effect of Atmospheric Plasma Treatment on Mechanical Properties of 3D-Printed Continuous Aramid Fiber/PLA Composites

**DOI:** 10.3390/polym17030397

**Published:** 2025-02-01

**Authors:** Fidan Bilir Kilinc, Ebru Bozaci, Ahmet Cagri Kilinc, Turker Turkoglu

**Affiliations:** 1 Graduate School of Natural and Applied Sciences, Department of Textile Engineering, Ege University, 35100 Izmir, Turkey; fidanbilir@hotmail.com; 2 Department of Textile Engineering, Ege University, 35100 Izmir, Turkey; ebru.bozaci@ege.edu.tr; 3 Department of Mechanical Engineering, Osmaniye Korkut Ata University, 80010 Osmaniye, Turkey; 4 Department of Mechanical Engineering, Balıkesir University, 10100 Balıkesir, Turkey; turker.turkoglu@balikesir.edu.tr

**Keywords:** aramid, 3D printing, composite, continuous fiber, atmospheric plasma treatment, FDM

## Abstract

In this study, an aluminum heating block with two inlets (for the Polylactic acid (PLA) filament and the continuous aramid fiber) was produced and placed onto an extruder, and continuous-aramid-fiber-reinforced PLA composites were fabricated by using the nozzle impregnation method. Layer height values of 0.4 mm, 0.6 mm, and 0.8 mm and hatch spacing values of 0.6 mm, 0.8 mm, and 1.0 mm were used for the investigation of the processing parameters on the properties of composites by differentiating the reinforcement volume fraction. Additionally, atmospheric plasma treatment was used for the surface modification of the reinforcement fiber. The properties of composites reinforced by using surface-modified fibers were also investigated in order to reveal the efficacy of the atmospheric plasma treatment on the properties of composites. The effect of the atmospheric plasma treatment on the fiber properties was investigated by using scanning electron microscopy (SEM), Fourier-transform infrared spectroscopy (FT-IR), and X-ray photoelectron spectroscopy (XPS). Continuous-aramid-fiber-reinforced PLA composites were characterized mechanically by fiber pull-out, tensile, and flexural testing. The fracture surfaces of composites were analyzed by using SEM. The combination of a reduced layer height and a narrower hatch spacing yielded the best mechanical performance, with a tensile strength of 410.25 MPa achieved at a 0.6 mm layer height and a 0.4 mm hatch spacing. This combination minimizes void formation, enhances fiber alignment, and strengthens interlayer adhesion, leading to superior mechanical properties. The FTIR and XPS results showed that atmospheric plasma modification can enhance the interfacial bonding strength by improving the surface morphology and increasing the content of polar groups on the fiber surface. By combining optimized manufacturing conditions with the atmospheric plasma treatment, the mechanical performance of continuous-aramid-fiber-reinforced PLA composites was enhanced.

## 1. Introduction

Additive manufacturing (AM), commonly referred to as 3D printing, has revolutionized the manufacturing landscape by enabling the production of complex geometries with minimal material waste and an increased customization potential [1,2,3]. Unlike traditional manufacturing methods, AM allows layer-by-layer construction, offering significant advantages such as reduced lead times, enhanced design flexibility, and lower tooling costs. However, challenges persist, including a limited material selection, anisotropic mechanical properties, and relatively slow production rates for large-scale applications. These drawbacks necessitate ongoing research into material innovations and process optimization in order to enhance the capabilities and performance of AM-produced components [4,5].

Polylactic acid (PLA) is a biodegradable thermoplastic polymer derived from renewable resources such as corn starch or sugarcane. Its environmental benefits, coupled with good processability, make PLA a popular choice in additive manufacturing. When reinforced with high-performance fibers like aramid, composites exhibit significant improvements in tensile strength, thermal stability, and energy absorption capabilities. Aramid fibers are renowned for their exceptional strength-to-weight ratio, high impact resistance, and thermal stability, making them ideal reinforcements for applications requiring a superior mechanical performance. Conversely, aramid fibers, owing to their high crystallinity, possess a low chemical reactivity and exhibit smooth surfaces. As this property can affect the performance of aramid fibers in composite materials, various modification methods have been investigated to strengthen the interfacial bond and increase the surface roughness. Among these methods, chemical and physicochemical treatments are prominent [6,7].

Secondary processing techniques, such as atmospheric plasma treatment, have been increasingly applied to enhance the interfacial bonding between reinforcement fibers and polymer matrices in AM composites. This surface modification method alters the chemical and physical properties of the fiber surface, improving wettability and adhesion. Such treatments are critical for optimizing the mechanical properties of composites, as they mitigate issues like delamination and poor stress transfer, which are common in non-modified-fiber-reinforced composites [8,9,10].

Hou et al. [11] developed a comprehensive model to analyze the mechanical behavior of 3D-printed continuous-fiber-reinforced composites (CFRCs) with varying fiber content. The study mapped the relationship between the fiber content and material properties, revealing the effects of porosity and fiber orientation on stiffness and strength. Failure mechanisms were examined under different loading conditions, showing enhanced fracture toughness through crack propagation along fibers. The model provides a framework for optimizing functionally graded CFRCs, supporting their application in high-performance industries such as the aerospace and automotive industries. This research underscores the critical role of fiber content in improving mechanical performance and optimizing manufacturing processes. Building on the understanding of continuous-fiber-reinforced composites, the recyclability of materials in 3D printing is gaining attention due to its potential for enhancing the sustainability and lifecycle of composites. Wei et al. [12] demonstrated that recycling and reprocessing can not only recover the structural integrity of composites but also, in some cases, improve their mechanical properties. For example, the tensile strength of recycled-continuous-Kevlar-fiber-reinforced PLA composites (CKF/PLA) surpassed that of virgin materials due to the better fiber impregnation during reprocessing. The study attributed these improvements to the refinement of the fiber–matrix interface and the enhanced alignment of fibers, which contribute to more efficient stress transfer. Furthermore, the reduced degradation of recycled composites compared to their virgin counterparts during aging highlights the role of recycling in mitigating environmental stressors. These findings underline that, beyond environmental benefits, recycled materials in composites can exhibit superior or comparable mechanical properties, emphasizing the dual advantages of economic efficiency and material performance enhancement. Expanding the scope of material reinforcement, Rijckaert et al. [13] explored the production of continuous-aramid-fiber-reinforced PETG composites with high fiber loading using the Fused Filament Fabrication (FFF) method. Through in-nozzle impregnation and adjustments to the printing parameters, they achieved fiber volume fractions ranging from 20% to 45%. The study demonstrated significant mechanical improvements, with tensile modules increasing from 2.2 GPa for unreinforced PETG to 33 GPa in reinforced composites. Additionally, detailed analyses of the microstructure and void content revealed how printing parameters influenced performance. These findings suggest that 3D-printed composites can rival traditional manufacturing methods in mechanical performance, offering innovative approaches for material design. Focusing on the optimization of fiber-reinforced composites, Ojha et al. [14] examined the mechanical properties of 3D-printed continuous-Kevlar-fiber-reinforced composites. Using onyx (a carbon fiber and nylon blend) as the matrix material, their study revealed that Kevlar fiber reinforcement enhanced tensile strength by up to 11 times and impact strength by up to 3 times. However, the study also identified challenges such as interlayer delamination at higher reinforcement ratios due to insufficient wetting by the matrix. These findings underline the importance of fiber orientation and content in achieving optimal performance while providing a roadmap for designing high-performance 3D-printed composites. Collectively, these studies demonstrate the transformative potential of continuous fiber reinforcement in 3D-printed composites. Unlike the existing studies, our research incorporates atmospheric plasma treatment as a pre-processing method to enhance the fiber–matrix interfacial bonding. This innovative approach is expected to significantly improve mechanical performance by addressing interfacial weaknesses, thereby advancing the application potential of 3D-printed continuous-fiber-reinforced composites.

This study contributes to the field of additive manufacturing by integrating continuous aramid fibers into PLA composites using a nozzle impregnation method. The experimental approach includes variations in the layer height and hatch distance to investigate their impact on the reinforcement volume fraction and composite performance. The use of atmospheric plasma treatment to modify the surface of aramid fibers represents a novel intervention aimed at addressing long-standing challenges in fiber–matrix interfacial bonding, such as delamination and weak adhesion. Unlike conventional surface treatments, atmospheric plasma treatment is a rapid, environmentally friendly, and solvent-free method that alters the chemical and physical properties of the fiber surface, promoting stronger interfacial interactions. Studies on the 3D fabrication of continuous-aramid-fiber-reinforced composites in the literature have focused on investigating the effects of the printing parameters, the repetitive use of fibers, or the geometric structure on composite properties. However, there is a large gap in investigating the effects of the fiber modification on composite properties. In this context, this study aims to fill this gap with atmospheric plasma treatment, an environmentally friendly surface modification. Previous studies have not systematically explored the effects of atmospheric-plasma-treated aramid fibers on the properties of PLA-based 3D-printed composites. This method significantly improves fiber wettability, promotes uniform stress distribution, and mitigates interfacial defects, which collectively contribute to the durability and reliability of the composites under mechanical loading. Furthermore, this study uniquely combines optimized additive manufacturing parameters—such as a reduced layer height and a narrower hatch spacing—with advanced surface treatment techniques in order to achieve an enhanced mechanical performance. By addressing the interplay between the process parameters and post-processing modifications, this research bridges a critical gap in the literature and provides a comprehensive understanding of the structural and functional optimization of 3D-printed composites. By characterizing these composites through tensile testing and analyzing fracture surfaces via SEM, this study not only reveals the efficacy of atmospheric plasma treatment but also provides a framework for future applications of continuous-fiber-reinforced PLA composites in high-performance engineering domains, including the aerospace, automotive, and defense industries. 

## 2. Materials and Methods

### 2.1. Materials

Continuous 228dtex×3 S aramid yarn (AOYI Inc., Guangzhou, China), which is made by twisting three strands, was used as reinforcement fiber. The term continuous refers to the reinforcement fiber yarn rather than thermoplastic filament, which is matrix material.

Clear polylactic acid (PLA) filament (Esun Industrial Co., Ltd., Shenzhen, China), with a density of 1.23 g/cm^3^, melt flow index of 5 (190 °C/2.16 kg), and a diameter of 1.75 mm, was used as matrix material.

### 2.2. Surface Modification of Fibers

**Figure 1:**
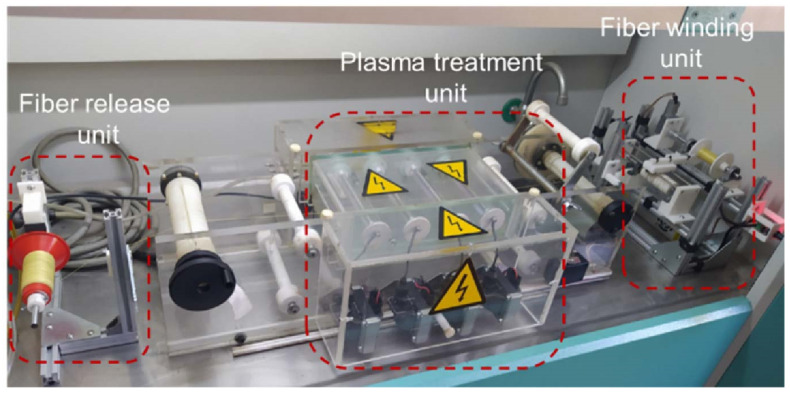
Lab-scale atmospheric plasma treatment system.

Lab-scale dielectric barrier discharge (DBD) plasma system operating under atmospheric conditions was utilized for surface modifications of aramid fibers. Lab-scale treatment system is shown in Figure 1. The plasma system was explained in detail elsewhere [15]. The plasma system is capable of working under both argon and air as purge gas. In this study, air was used as a purge gas. The aramid fibers were continuously passed through the system and treated at discharge power of 200 W for treatment duration of 12 s, and, finally, collected onto a spool. Spool of atmospheric-plasma-treated fiber was then placed onto 3D printer for printing process.

### 2.3. 3D Printing of Continuous-Aramid-Fiber-Reinforced PLA Composites

**Figure 2:**
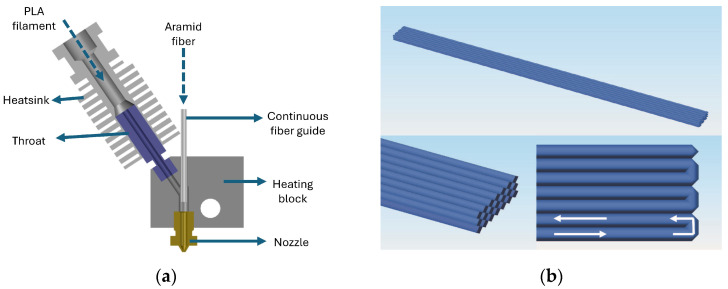
Schematic representation of (**a**) hot-end system; and (**b**) continuous printing path for unidirectional composites (white arrows represent the nozzle path).

A heating block was produced for the 3D printing process of continuous-aramid-fiber-reinforced PLA composites. The reinforcement continuous fiber and thermoplastic matrix were fed into the system from separated points. By providing the melt thermoplastic infusion to the continuous aramid fiber in the heating block, continuous-aramid-reinforced PLA matrix composite lines were deposited from the nozzle. Schematic representation of hot-end system for 3D printing of continuous aramid fiber/PLA composites is shown in Figure 2a.

In the first part of the study, the printing parameters of hatch spacing and layer thickness were optimized by using non-modified aramid fibers. After optimization, composites were 3D-printed by using atmospheric-plasma-treated aramid fibers to investigate the effect of atmospheric plasma treatment on the mechanical properties of the composites, non-modified. The printing parameters are shown in Table 1, and continuous printing path for unidirectional composites is shown in Figure 2b. Printing speed and printing temperature of unidirectional composites were fixed at 10 mm/s and 210 °C, respectively, during 3D printing process of all specimens.

### 2.4. Characterization of Fibers

The effect of atmospheric plasma treatment on the surface morphology of aramid fibers was investigated by using scanning electron microscope (SEM; ZEISS, EVO HD15, Oberkochen, Germany). Fibers were coated by Au-Pd for 60 s by using sputter coater to prevent charging problems during SEM investigations. The coating thickness was ~6 nm. The Fourier-transform infrared spectrometer (Perkin Elmer Spectrum BX, Waltham, MA, USA) was used for characterization of surface functional groups of fibers. The spectra of the fibers were obtained in the range of 650–4000 cm^−1^ wavenumber with a scan rate of 20 scans per minute at a resolution of 2 cm^−1^. The changes on the surface elemental compositions of the aramid fibers were determined by X-ray photoelectron spectroscopy (XPS). XPS analyses was performed using a Thermo Scientific K-Alpha XPS (Thermo Fisher Scientific, Horsham, UK) spectrometer equipped with an Al Kα X-ray radiation. The X-ray spot size and the step size were both set to 250 μm. A survey spectrum with a pass energy of 200 eV and snapshot scans with a pass energy of 1 eV were collected during the mapping.

### 2.5. Characterization of Composites

**Figure 3:**
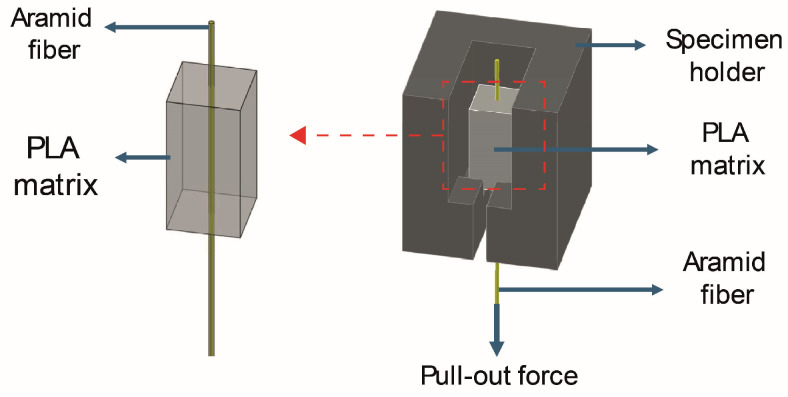
Schematic representation of pull-out sample and test setup (Red box and arrow indicate isolation of specimen).

Fiber volume fractions of 3D-printed continuous aramid fiber composites were determined by using cross-sectional microstructural images [16]. The effect of atmospheric plasma treatment on fiber–matrix interface bonding was investigated by fiber pull-out test. For this purpose, unreinforced samples with dimensions of 5 mm × 10 mm × 5 mm (printing directions: x × y × z) were printed. When half height of the sample was printed, aramid yarns were placed in the middle of the sample straightly and the printing process was continued as proposed by Nuhoglu et al. [17]. Schematic representations of pull-out test samples are shown in Figure 3. Ten specimens were tested with a crosshead speed of 1 mm/min.

**Figure 4:**
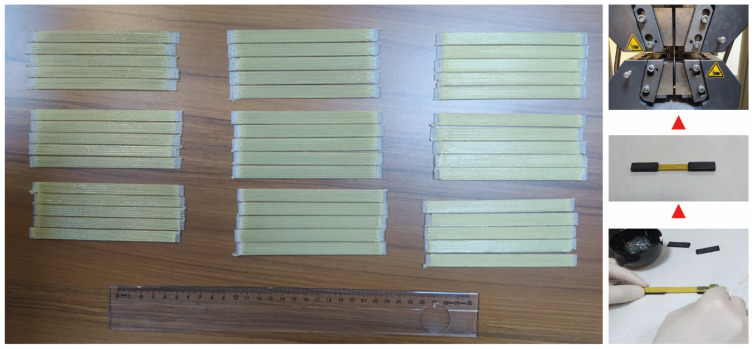
3D-printed composite specimens and preparation of tensile testing specimen.

The tensile strength of composites was determined by tensile testing according to ASTM 3039 [4]. Unidirectional specimens with dimensions of 130 × 12 × 2.4 mm (x × y × z) were printed for tensile testing. Then, 3D-printed tabs were attached at both ends of the specimen to provide better load transfer and reduce stress concentrations at the grips. Next, 3D-printed composite specimens and preparation of tensile testing specimens are shown in Figure 4. Fracture surfaces of tensile test specimens were investigated by using scanning electron microscope (SEM; ZEISS, EVO HD15, Germany).

Flexural properties of continuous-aramid-fiber-reinforced PLA composites were determined by using three-point bending test. Three-point bending tests were performed by using according same specimens of to ASTM D760 [10]; specimens were simply supported at two points and loaded at the midpoint with a crosshead speed of 1.3 mm/min and span-to-depth ratio of 20:1.

## 3. Results and Discussion

### 3.1. Fiber Characterization

**Figure 5:**
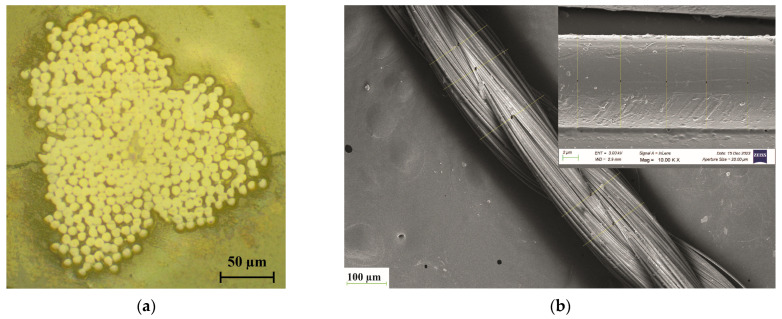

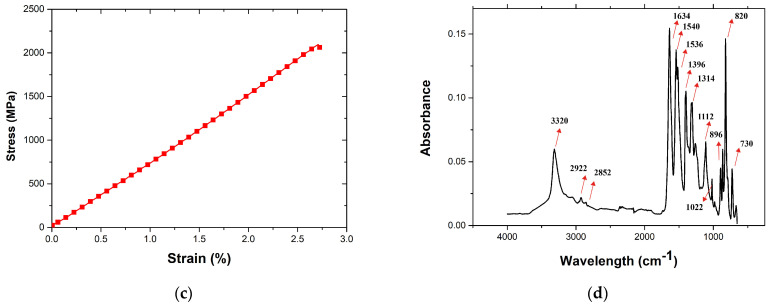
(**a**) Cross-sectional image; (**b**) SEM images; (**c**) tensile stress–strain curve, and (**d**) FT-IR spectra of aramid yarn.

The cross-sectional image, SEM images, tensile stress–strain curve, and FT-IR spectra of aramid yarn are shown in Figure 5a–d. A piece of aramid strand was cut and immersed in epoxy resin for the optical microscope investigation. After the curing of the epoxy resin, the specimen was ground by using SiC paper of 240 to 1200 grit under water. Finally, the ground surface of the specimen was polished by using polishing paste containing 6 µm of synthetic diamond particles. As seen from the cross-sectional image (see Figure 5a), the aramid strand is made by twisting three strands. The yarn contains 420 fibers with a fiber diameter of 12.24 µm, which is measured by using an SEM image of the fiber (see Figure 5a,b). The diameter of the yarn is measured as 193.18 µm. The stress–strain curve of the aramid yarn is shown in Figure 5c. The tensile strength of the yarn is determined as 169 cN/tex. The FT-IR graph of the pristine aramid yarn obtained with a wavenumber in the range of 650–4000 cm^−1^ is shown in Figure 5d. The peak located at 3320 cm^−1^ indicates the existence of –NH groups derived from the stretching vibration on the amide bond [18,19,20]. The peaks located at 2922 cm^−1^ and 2852 cm^−1^ are derived from the stretching vibrations of –CH– and –CH_2_–, respectively [18,21]. The sharp peak located at 1634 cm^−1^ corresponds to the C=O stretching vibration peak of the amide I band [18,20]. The peaks located at 1540 cm^−1^ and 1536 cm^−1^ are attributed to the in-plane bending vibration of the N–H group [18,20,21]. The peak located at 1396 cm^−1^ is attributed to the semicircle stretching of the free hydrogen of the aromatic ring [22]. The peaks located at 1314 cm^−1^ and 1248 cm^−1^ are derived from the stretching vibration of the C–N of the amide II and amide III bands, respectively [20,23,24]. The peak located at 1112 cm^−1^ is attributed to the in-plane deformation mode of the –C–H bond [22]. The peak located at 1112 cm^−1^ is attributed to the vibration of the C–H bond of the benzene ring [22]. The peaks located at 896 cm^−1^ and 730 cm^−1^ are attributed to the out-of-plane deformation modes of the N–H group. The peak located at 820 cm^−1^ is derived from the out-of-plane deformation mode of the –C–H bond [15,22].

### 3.2. Composite Characterization

**Figure 6:**
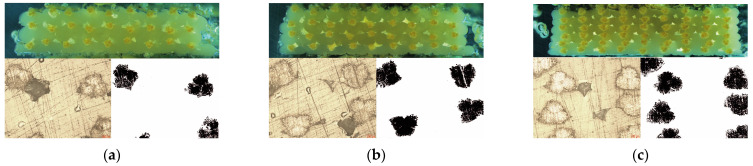

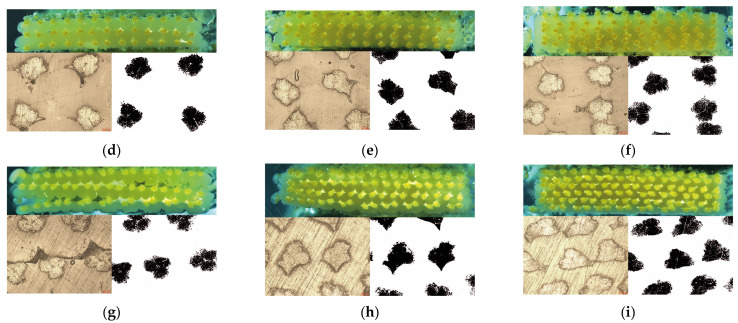
Cross-sectional images of (**a**) 1-08; (**b**) 1-06; (**c**) 1-04; (**d**) 08-08; (**e**) 08-06; (**f**) 08-04; (**g**) 06-08; (**h**) 06-06; and (**i**) 06-04.

The cross-sectional morphologies of continuous aramid fiber/PLA composites 3D-printed with different parameters are shown in Figure 6a–i. In each figure, the stereo microscope image (top), optical microscope image (bottom left), and fiber fraction images obtained using ImageJ software (V 1.24) are shown. The reinforcing fibers are located close to each other, as expected with decreasing hatch spacing. A similar result was observed for decreasing layer thickness. With decreasing layer thickness and hatch spacing, the amount of reinforcement fibers deposited per unit area increased. The fiber volume fractions calculated by using optical microscope cross-sectional images are given in Table 2. As the hatch spacing and layer height values decreased from 1 mm and 0.8 mm to 0.6 mm and 0.4 mm, the fiber volume fraction value of the composites increased from 11.297 vol.% to 26.851 vol.%, respectively. As seen in Figure 6, fibers are located at the top of the deposited individual lanes rather than being centered. Ibrahim et al. [25] indicated that the force exerted by the moving of the printing head and nozzle causes tension on the fiber and this situation results in the non-aligned settlement of the reinforcement fiber [25]. In addition, gaps originating from the 3D printing process are observed at the contact points of the deposited individual lines in the cross-sections. Especially in the 06-08 sample, the gap between the layers is noticeable. The layer thickness being higher than the hatch spacing value resulted in the delamination of the layers. It is clearly seen that the gaps originating from the 3D printing became smaller with decreasing layer thickness and hatch spacing values [26,27].

**Figure 7:**
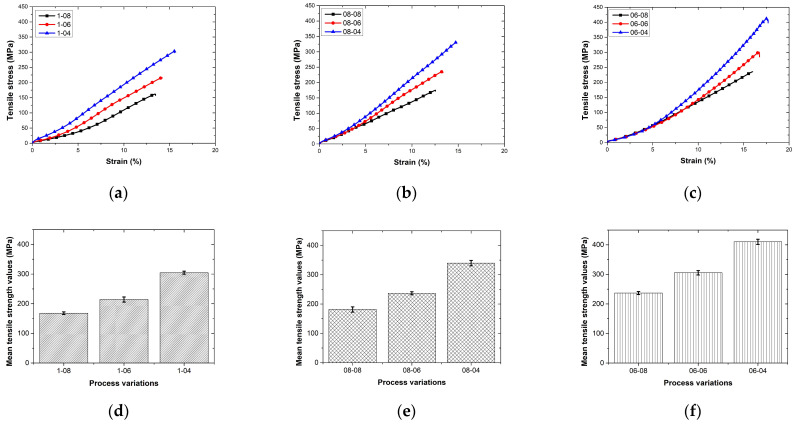
Stress–strain curves (**a**–**c**) and mean tensile strength values (**d**–**f**) of specimens printed with different layer height and hatch spacing values.

The stress–strain curves and mean tensile strength values of specimens printed with different layer height and hatch spacing values are shown in Figure 7a–f. The mechanical performance of continuous-aramid-fiber-reinforced PLA composites demonstrates the critical influence of additive manufacturing parameters, such as the layer height and hatch spacing, on the tensile strength. These findings underscore the importance of parameter optimization in tailoring the mechanical properties for advanced engineering applications. By analyzing the effects of individual parameters and their combined impact, this study provides a comprehensive understanding of how to achieve optimal composite performance.

The tensile strength of the composites increased markedly with decreasing layer height. At a constant hatch spacing of 0.4 mm, the tensile strength improved from 304.87 MPa at a layer height of 1.0 mm to 410.25 MPa at 0.6 mm. This improvement can be attributed to the enhanced interlayer bonding achieved with thinner layers, which provide a greater overlap between successive layers during the printing process. A thinner layer thickness increases the mechanical strength by enhancing the fiber–matrix interaction. In the study of Hou et al. [28], it was reported that this effect was similarly observed on the compressive strength of continuous-Kevlar-fiber-reinforced PLA composites. The resulting denser composite structure minimizes void formation and enhances stress transfer efficiency. Moreover, Jiang et al. [29] reported that a certain layer thickness gives the best results in tensile strength in continuous-aramid-fiber-reinforced nylon 12 composites, and it was stated that lower layer thicknesses improve interlayer bonding and minimize pore formation. However, it was also emphasized that very low layer thicknesses may lead to the uneven distribution of the resin around the fibers, which may adversely affect mechanical performance. Furthermore, smaller layer heights enable more precise fiber placement, promoting uniform stress distribution and reducing the likelihood of stress concentration zones. This trend highlights the significance of the layer height as a primary parameter in improving the composite strength. However, layer height alone does not dictate mechanical performance; the hatch spacing must also be considered in order to achieve an optimized structure.

On the other hand, a narrower hatch spacing significantly contributed to a higher tensile strength, complementing the benefits of a reduced layer height. For instance, at a layer height of 0.6 mm, reducing the hatch spacing from 0.8 mm to 0.4 mm resulted in an increase in tensile strength from 237.09 MPa to 410.25 MPa. A narrower hatch spacing promotes material continuity by increasing the overlap between deposition lines, thereby reducing the porosity and enhancing the fiber–matrix interface. This ensures better load transfer and mechanical stability within the composite. The interplay between the hatch spacing and layer height becomes evident when both parameters are optimized simultaneously, emphasizing the need for a holistic approach to parameter selection in additive manufacturing. The effect of hatch spacing on mechanical properties has been frequently emphasized in the literature. For example, Hou et al. [30] reported that, by optimizing the hatch spacing, superior stability and a homogeneous force distribution were achieved in continuous-fiber-reinforced energy absorption tubes. In particular, it has been stated that a 0.4 mm hatch spacing plays a critical role in improving mechanical performance. Similarly, in our study, narrower hatch spacing values were observed to increase the tensile strength, and it was confirmed that optimizing this parameter has a significant effect on the strength.

**Figure 8:**
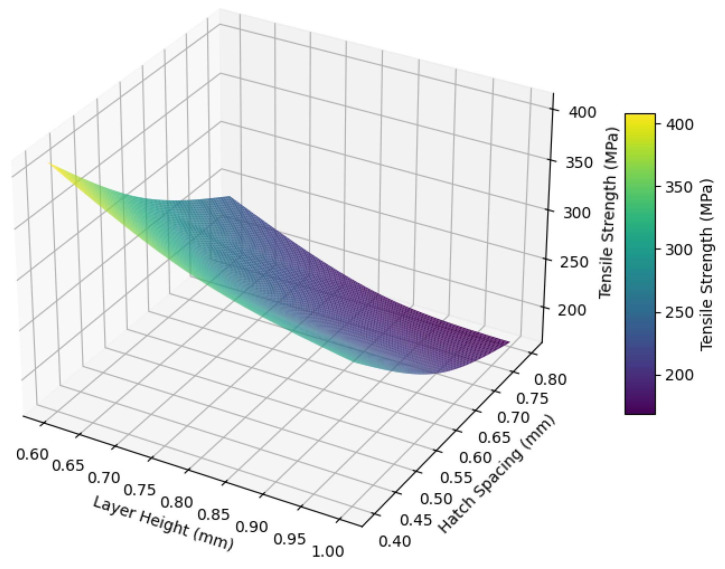
The combined effect of layer height and hatch spacing on tensile strength.

The combination of a reduced layer height and a narrower hatch spacing is shown in Figure 8. The best mechanical performance, with a tensile strength of 410.25 MPa, is achieved at a 0.6 mm layer height and a 0.4 mm hatch spacing. This combination minimizes void formation, enhances fiber alignment, and strengthens interlayer adhesion besides increasing fiber fraction, leading to superior mechanical properties. In contrast, the combination of larger layer heights and wider hatch spacing, such as 1.0 mm and 0.8 mm, resulted in the lowest tensile strength of 168.12 MPa, highlighting the detrimental effects of poor layer bonding and excessive void formation. In this study, the effectiveness of narrower hatch spacing and thinner layer height parameters in increasing tensile strength is in agreement with the findings of Rijckaert et al. [13]. The results obtained confirm that optimized printing parameters are an important factor in improving mechanical performance.

**Figure 9:**
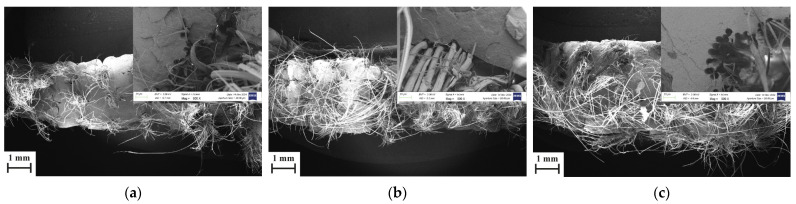

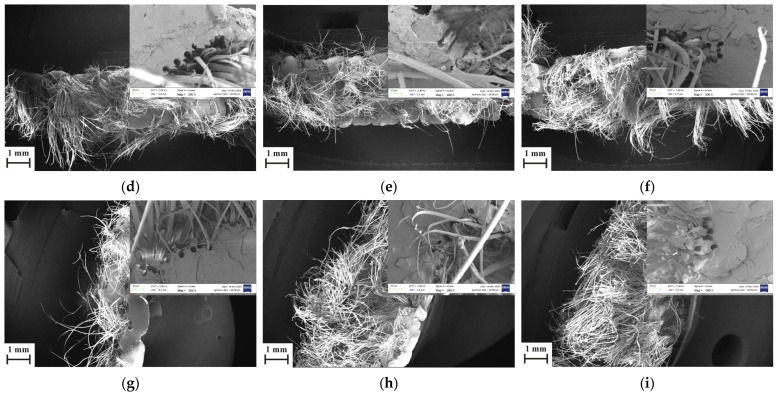
Fracture surface SEM images of (**a**) 1-08; (**b**) 1-06; (**c**) 1-04; (**d**) 08-08; (**e**) 08-06; (**f**) 08-04; (**g**) 06-08; (**h**) 06-06; and (**i**) 06-04.

The SEM images of the failure surfaces of the samples after the tensile test are shown in Figure 9a–i. Similar failure mechanisms were observed for the analyzed fracture surfaces of the different specimens. The increase in the number of fibers is clearly seen with the decrease in the hatch spacing value and the decrease in the layer thickness value. Regardless of the printing parameters, all samples exhibited failure behavior in the form of ductile fracture with necked fibers, which is a typical fracture characteristic of the aramid fibers [31,32]. Another situation that can be stated as common for all samples is that the matrix penetration into the fibers is limited and the matrix penetration during the nozzle impregnation process is limited to the outer surface area of the yarn. In these regions, it is seen that the fibers which are surrounded by little or no matrix are pulled out from the matrix due to the weak fiber–matrix interfacial bonding.

**Figure 10:**
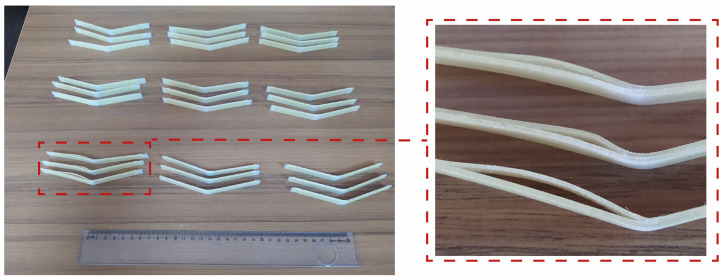
Bending test samples.

The flexural strength results of continuous-aramid-fiber-reinforced PLA composites were evaluated under varying process parameters, including the layer thickness (0.4 mm, 0.6 mm, and 0.8 mm) and hatch spacing (1.0 mm, 0.8 mm, and 0.6 mm). These parameters significantly influenced the mechanical performance of the composites. Three specimens were produced for each set of production parameters for bending tests. After the bending test, the deformed samples are shown in Figure 10. It should be noted that no fracture was observed in the samples. It is clearly seen that delamination was observed in the 06-08 samples. The layer thickness being higher than the hatch spacing value resulted in the delamination of the layers.

**Figure 11:**
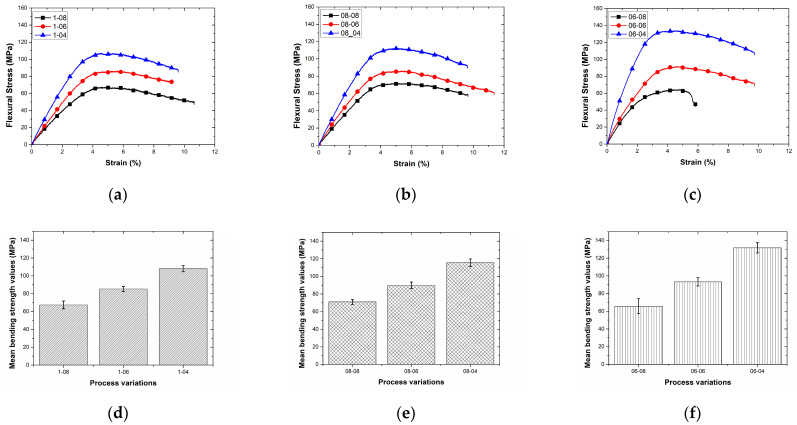
Bending stress–strain curves (**a**–**c**) and mean bending strength values (**d**–**f**) of specimens printed with different layer height and hatch spacing values.

Flexural stress–stain curves and a bar chart of the mean strength values are shown in Figure 11a–f. For composites printed with a layer thickness of 0.8 mm, the flexural strength ranged from 65.73 ± 8.57 MPa to 70.98 ± 2.88 MPa, showing relatively lower values compared to other layer thicknesses. This trend can be attributed to the reduced interlayer bonding and higher void formation observed at an increased layer thickness. The lowest flexural strength was recorded at a hatch spacing of 0.6 mm, likely due to the insufficient overlap between deposition lines at this parameter combination.

### 3.3. Effect of Atmospheric Plasma Treatment

When the layer thickness was reduced to 0.6 mm, the flexural strength improved significantly, ranging from 85.30 ± 2.79 MPa to 93.27 ± 4.55 MPa. The enhancement can be associated with an improved layer fusion and a reduced void content, providing better stress transfer during bending. The composites fabricated with the smallest layer thickness of 0.4 mm exhibited the highest flexural strength, ranging from 107.99 ± 3.40 MPa to 131.67 ± 5.79 MPa. The superior performance at this parameter is attributed to the enhanced interlayer adhesion and improved fiber alignment, which collectively minimize the stress concentration zones and maximize the mechanical load distribution. Among all configurations, the highest flexural strength was achieved at a layer thickness of 0.4 mm and hatch spacing of 0.6 mm, suggesting this combination as the optimal parameter set for maximizing flexural performance.

Overall, these results emphasize the importance of optimizing additive manufacturing parameters to tailor the mechanical properties of continuous-fiber-reinforced composites for specific applications. The interplay between the layer thickness and hatch spacing demonstrated a clear influence on the flexural strength, highlighting the critical role of process parameter optimization in achieving high-performance composite structures.

The bending strength results of the composites fabricated with the 06-04 production parameters (0.6 mm layer height and 0.4 mm hatch spacing) demonstrated superior mechanical performance compared to other tested configurations. Specifically, the bending strength achieved for the 06-04 configuration without surface modification was 133.77 MPa, marking it as the most optimized set of parameters in terms of the layer height and hatch spacing. These results highlight the critical role of a tighter layer stacking and narrower hatch spacing in enhancing interlayer adhesion and reducing void formation, which collectively contribute to improved bending performance.

**Figure 12:**
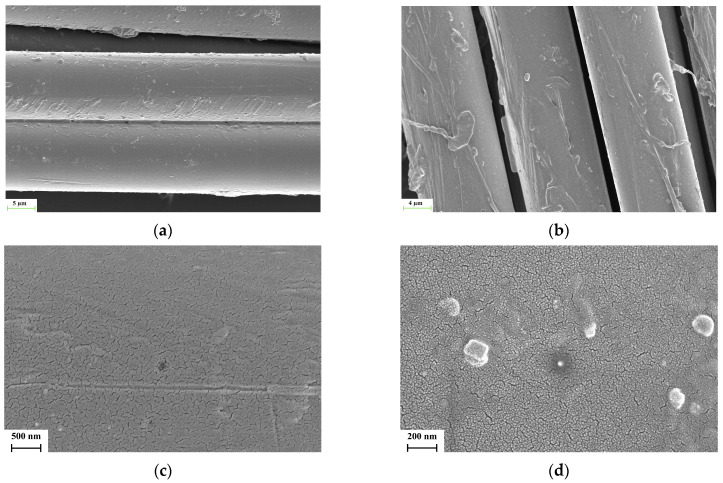
SEM micrographs of (**a**,**c**) non-modified and (**b**,**d**) atmospheric-plasma-treated aramid fibers.

SEM micrographs which are given in Figure 12a–d show the morphology of non-modified and treated aramid fibers, respectively. A relatively clean and smooth surface was observed for non-modified fibers (see Figure 12a,c) while the atmospheric-plasma-treated fiber has an uneven surface (see Figure 12b,d). Surface etching and roughening are clearly visible in the aramid fiber following plasma treatment which is caused mostly by the bombardment of charged particles [33]. The surface of fibers was partially etched by the bombardment of intense electrons and ions during plasma treatment. As is indicated in the literature, the etching causes the roughening and improved adhesion between the fiber and matrix, which can be formed by the enhanced surface energy and improved mechanical properties of composites, which can be achieved by the strong mechanical interlocking of the fibers and matrix [33,34,35].

**Figure 13:**
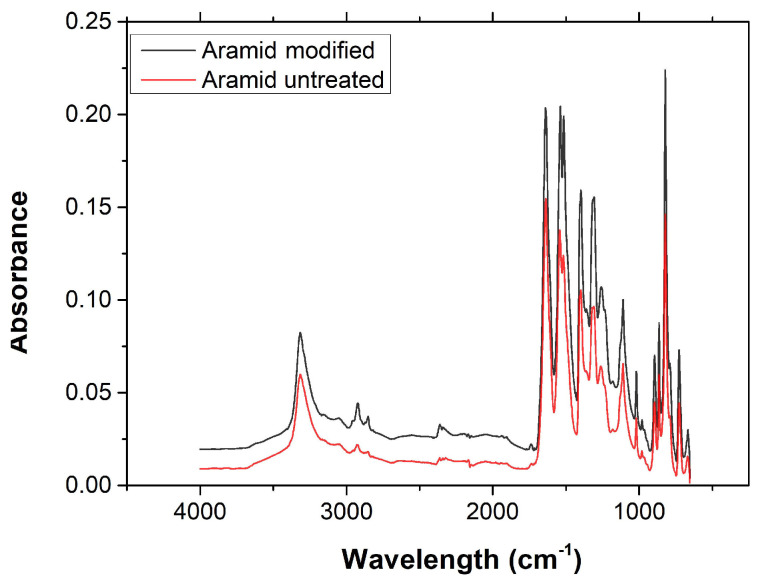
FT-IR spectra of non-modified and plasma-treated aramid fibers.

The FT-IR spectra of modified and non-modified aramid fiber are given in Figure 13. The fact that no new peaks appeared suggests that the treatment may have primarily altered the existing functional groups on the fiber surface rather than introducing entirely new functional groups. And the chemical structure of plasma-treated aramid fiber did not change obviously when compared with the non-modified fiber sample, which demonstrates that atmospheric plasma treatment did not break the fiber bulk structure. The increased peak intensity at 1737 cm^−1^, which is the characteristic absorption peak of the stretching vibration of C=O in the carboxyl group, was attributed to the number of -COOH groups on the fiber surface, which was increased by plasma treatment [36]. Jia et al. [23] indicated that the oxidation effect can be observed during plasma treatment which is confirmed by the increased oxygenic group on the surface. Polar bonds such as C=O and C–O are related to the improved wettability and increased surface energy [35]. Besides oxygenic groups, the intensities of peaks related to N–H and C–N increased noticeably [23].

The surface chemical compositions of treated and untreated aramid fibers were obtained by the XPS technique and the surface elemental composition results are given in Table 3. The data revealed an increase in both the O_1S_/C_1S_ and N_1S_/C_1S_ ratios from 0.25 to 0.27 and from 0.016 to 0.019, respectively, following plasma treatment. The data revealed an increase in both the O_1S_/C_1S_ and N_1S_/C_1S_ ratios from 0.25 to 0.27 and from 0.016 to 0.019, respectively, following plasma treatment. These findings suggest that atmospheric air plasma treatment induces the formation of additional oxygen-containing functional groups, such as carboxyl and hydroxyl groups on the aramid fiber surface. These polar groups on the surface increase the hydrophilicity of the fibers, reducing the water contact angle. This can be attributed to the morphological changes induced by the plasma on the fiber surface and the formation of hydrogen bonds by the polar groups. The obtained results suggest that atmospheric plasma treatment can modify the surface properties of aramid fibers, enhancing the interfacial adhesion in composite materials and making them suitable for various applications.

Table 4 presents the deconvoluted C_1S_ core-level spectra of both untreated and plasma-treated aramid fibers. The deconvolution of the C_1S_ signal revealed three primary peaks: C-C/C-H (284.54 eV), C-O/C-O-C (286.15 eV), and O-C=O (288.52 eV). In untreated aramid fibers, the relative peak areas for C-C/C-H, C-O/C-O-C, and O-C=O were 75.0%, 19.2%, and 5.8%, respectively, yielding a polar-to-nonpolar group ratio of 0.33. Following atmospheric plasma treatment, the C-C/C-H peak area decreased to 70.8% while the relative abundance of polar groups increased to 29.2%, resulting in an elevated polar-to-nonpolar group ratio of 0.41. This observed decrease in the C-C/C-H peak area ratio, coupled with an increase in the C-O peak area, suggests an enhanced surface oxidation of the fibers. This oxidation, likely induced by ions, electrons, and UV radiation generated within the plasma, is expected to improve the adhesion properties of aramid fibers by modifying their surface chemistry, potentially reducing the chemical inertness and increasing the surface free energy.

**Figure 14:**
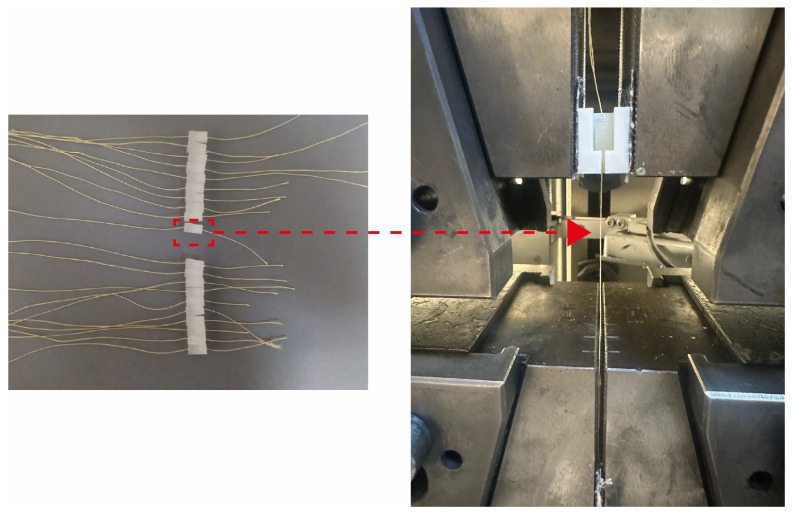
3D-printed pull-out test samples and pull-out test setup.

The 3D-printed pull-out test samples and pull-out test setup are shown in Figure 14. As seen in the figure, 3D-printed samples were again placed in the 3D-printed sample holder and the test was performed by pulling the aramid fiber from the tip.

**Figure 15:**
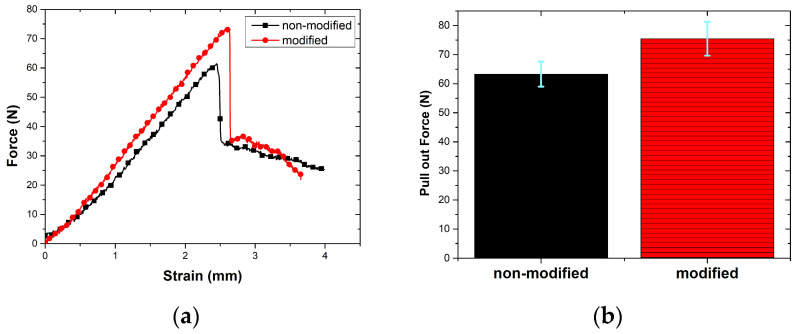
(**a**) Load–displacement curve, and (**b**) bar chart of peak forces.

The load–displacement curve was recorded during the test and this curve is shown in Figure 15a and the mean force values are shown in Figure 15b. As seen in the curves, typical pull-out behavior is clearly seen for both the raw and modified fibers. As the aramid fiber was pulled from the tip, the load increase was observed. The load peaked and then suddenly decreased. At this point, the fibers were detached from the matrix. After this point, the load value exhibited a decreasing behavior at a lower value. This behavior was observed due to the friction between the stripped fiber and the matrix [37]. It is obvious that the matrix-fiber detachment peak load value increases in atmospheric-plasma-treated fibers. While the non-modified fiber pull-out load value was measured as 63.28 ± 7.31 N, the pull-out load value of the atmospheric-plasma-treated fiber was measured as 75.43 ± 6.81 N. This can be attributed to both the improvement in the matrix-bond by the change in functional groups on the surface after plasma treatment and the mechanical interlocking between the matrix and the roughened fiber surface.

**Figure 16:**
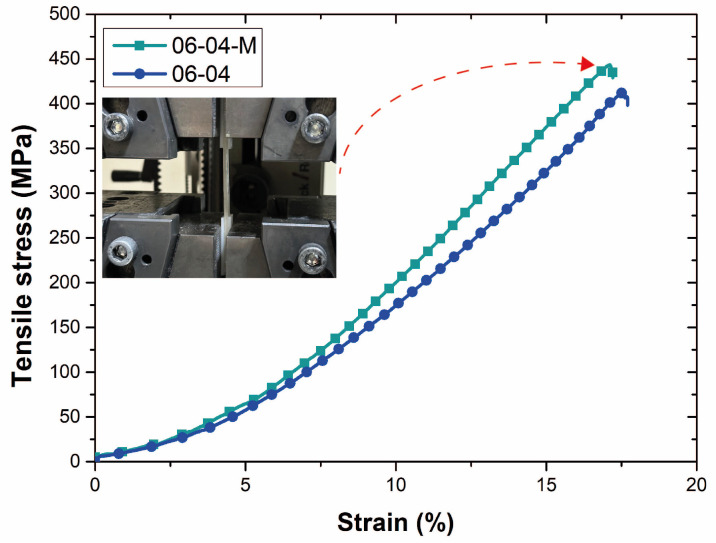
Stress–strain curves of non-modified and atmospheric-plasma-modified aramid fibers (Red arrow indicates curve of modified fiber).

The stress–strain curves of non-modified and atmospheric-plasma-modified aramid fibers are shown in Figure 16. The mechanical performance of the 06-04 specimen, fabricated under optimized additive manufacturing parameters, serves as a reference point for understanding the effects of surface treatments on the tensile strength. The non-modified 06-04 specimen exhibited a tensile strength of 410.25 MPa, showcasing the effectiveness of a reduced layer height and hatch spacing in achieving superior fiber alignment and enhanced interfacial bonding within the composite matrix. These results highlight the significance of parameter optimization in additive manufacturing for continuous-fiber-reinforced composites.

In comparison, the atmospheric-plasma-treated 06-04 specimen achieved a significantly higher tensile strength of 442.78 MPa, representing a notable improvement of approximately 7.83%. This enhancement can be attributed to the atmospheric plasma treatment’s ability to modify the surface properties of the aramid fibers, improving the wettability and interfacial adhesion between the reinforcement and the PLA matrix. The treatment promotes the formation of functional groups on the fiber surface, as verified by FTIR facilitating a stronger chemical interaction at the fiber–matrix interface. The superior performance of the treated specimen underscores the critical role of secondary surface treatments in overcoming the interfacial bonding limitations inherent in additively manufactured composites. While the optimized layer height and hatch spacing ensure an efficient mechanical load transfer through the aligned fibers, the plasma treatment further mitigates the potential stress concentrations and delamination at the fiber–matrix interface. Consequently, the mechanical integrity of the composite under tensile loading is significantly enhanced. Bertin et al. [38] reported that plasma treatment promotes the formation of free radicals on the surface, facilitating the addition of chemical groups to the polymer surface, which can contribute to the improvement of mechanical properties such as tensile strength. Similarly, Zarei et al. [39] suggested that atmospheric plasma treatment enhances interlayer bonding, positively impacting the mechanical performance. Additionally, Chen et al. [40] emphasized that the increase in surface energy after plasma treatment significantly strengthens the adhesion between fibers and the polymer matrix, further enhancing the composite’s mechanical characteristics.

The improvement in tensile strength of approximately 7.83% achieved through atmospheric plasma treatment demonstrates the potential of this method for enhancing the interfacial bonding in continuous-aramid-fiber-reinforced PLA composites. While this improvement may seem modest, its significance lies in the broader implications for long-term mechanical reliability and durability under repeated or extreme loading conditions. Atmospheric plasma treatment provides an environmentally friendly, solvent-free, and scalable solution that enhances fiber–matrix adhesion without altering the bulk properties of the fibers. This makes it particularly valuable for applications where interfacial bonding plays a critical role in performance, such as the aerospace and automotive sectors. However, we acknowledge that the additional processing cost associated with atmospheric plasma treatment could pose a challenge for widespread industrial adoption. Future research should aim to quantify the cost–benefit ratio more precisely by investigating the method’s impact on other critical performance metrics, such as fatigue resistance, impact strength, and thermal stability. Additionally, exploring process optimization, such as increasing the throughput of the plasma treatment system or integrating it directly into the additive manufacturing workflow, could help offset costs.

Regarding the limitations of this study, the inability of the PLA matrix to fully penetrate the fiber bundles due to the nozzle impregnation process is a critical aspect that warrants further exploration. This limitation likely restricts the effectiveness of the plasma treatment in reaching the inner fibers, which may reduce the overall mechanical benefits. Future studies could investigate alternative impregnation techniques or post-printing infiltration methods to enhance the matrix penetration and maximize the utilization of the treated fiber surface. Moreover, the use of alternative polymer matrices with lower viscosities or tailored rheological properties could improve fiber wetting and penetration, addressing the current limitations identified in this work. By discussing these limitations and opportunities, we aim to provide a roadmap for future research while emphasizing the significance of our findings in advancing the state of the art in continuous-fiber-reinforced composite materials.

**Figure 17:**
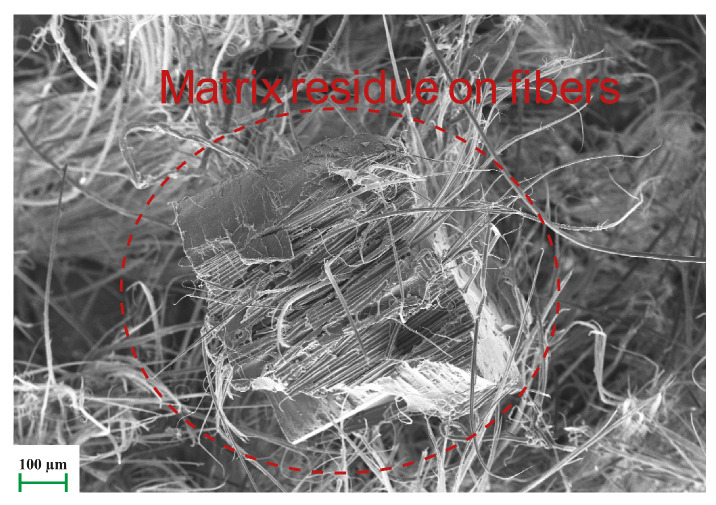
Tensile failure surface of atmospheric plasma modified sample (Red circle indicates matrix residue on fibers).

The SEM image of the damage surface of the atmospheric-plasma-treated fiber composite after the tensile test is shown in Figure 17. Although the fibers were damaged in the form of splitting and breaking similarly to the non-modified ones, the biggest difference here is that matrix residue was found on the fiber, which can be attributed to the improvement in the matrix-fiber interface bond with the plasma treatment.

To further enhance the mechanical properties of the 06-04 composite, an atmospheric plasma surface modification was applied to the reinforcement fibers prior to the additive manufacturing process. The surface-treated composite exhibited a significant improvement in bending strength, reaching a value of 157.42 MPa, representing an enhancement of approximately 17.67% compared to the non-modified composite. This improvement can be attributed to the atmospheric plasma treatment’s ability to modify the fiber surface by increasing surface roughness and introducing polar functional groups, as evidenced in prior studies.

The enhanced interfacial bonding between the aramid fibers and the PLA matrix, facilitated by atmospheric plasma treatment, contributes to a more efficient load transfer during bending stress. The treated fiber surface mitigates stress concentrations and reduces the likelihood of delamination or fiber pull-out, thereby improving the composite’s overall mechanical performance. Xu et al. [41], in their study, reported that atmospheric plasma treatment significantly enhances the surface properties of aramid fibers and promotes the formation of polar functional groups. This process improves the surface roughness of the fibers, thereby increasing their wettability and interfacial bonding strength. The researchers highlighted that the combined effects of chemical bonding and mechanical interlocking facilitate the more efficient stress transfer from the matrix to the fibers, ultimately improving the overall mechanical performance of the composites. In another study, Ye et al. [42] demonstrated that atmospheric pressure plasma treatment is highly effective for the surface modification of aramid fibers. The process was reported to enhance the surface morphology of the fibers and increase the density of polar functional groups on their surfaces. These improvements significantly elevated the surface energy and wettability of the fibers, resulting in stronger interfacial bonding with the epoxy matrix.

**Figure 18:**
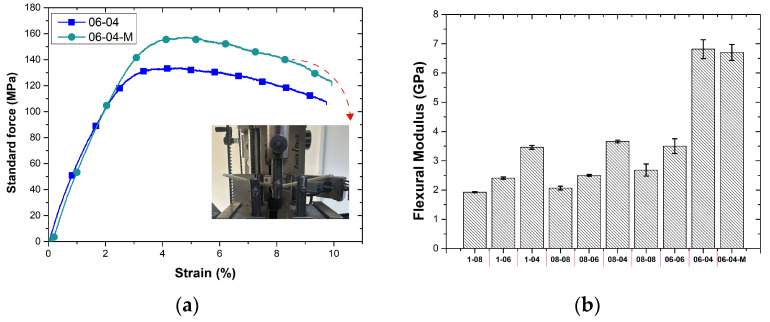
(**a**) Stress–strain curves of modified and non-modified bending test samples, and (**b**) flexural modulus of all samples (Red arrow indicates bending curve of modified composites).

These findings underscore the synergistic effect of optimized printing parameters and advanced surface modification techniques in achieving high-performance continuous-fiber-reinforced composites. While the optimized 06-04 parameters already deliver excellent results, the addition of atmospheric plasma treatment further pushes the boundaries of mechanical performance, making this approach particularly promising for high-stress applications in the aerospace, automotive, and structural engineering domains. The flexural stress–strain curves of modified and non-modified bending test samples and the flexural modulus of all samples are shown in Figure 18a and b, respectively. The atmospheric-plasma-treated 06-04-M sample, with a flexural modulus of 6.70 GPa, showed a comparable performance to the non-modified counterpart. This suggests that, while atmospheric plasma treatment enhances fiber–matrix adhesion through increased surface energy and improved chemical compatibility, the overall gain in the flexural modulus remains within a modest range. However, the standard deviation values indicate that the plasma-treated samples exhibited a more consistent performance, highlighting the process’s potential in achieving better reproducibility and reducing material variability.

Furthermore, samples with a larger hatch spacing and layer thickness, such as 1-08 and 08-08, displayed relatively lower flexural modulus values of 1.92 GPa and 2.06 GPa, respectively. This can be attributed to an increased void content and weaker interlayer adhesion, which limit the effective load transfer within the composite structure. The observed results align with previous findings in the literature, where optimizing printing parameters has been shown to significantly influence mechanical performance by minimizing defects and ensuring a uniform fiber distribution [42,43].

The results indicate a clear trend of an increasing flexural modulus with a decreasing layer thickness and hatch spacing. Among the tested samples, the 06-04 configuration exhibited the highest flexural modulus of 6.81 GPa, demonstrating the significant influence of optimized processing parameters on the mechanical performance of the composites. The reduction in layer thickness and hatch spacing resulted in improved interlayer bonding and enhanced fiber–matrix interaction, contributing to the superior flexural performance.

**Figure 19:**
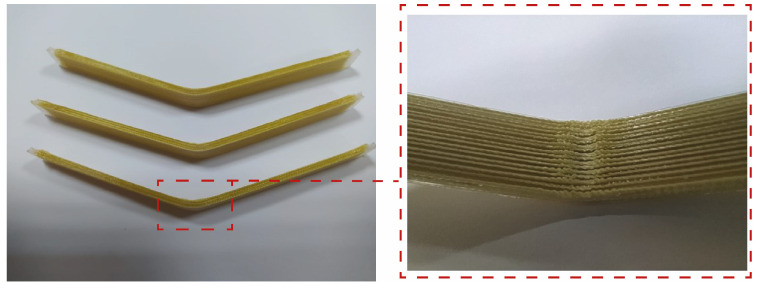
Failed specimens of 06-04-M after three-point bending test and compressive side of specimen.

Failed specimens of 06-04-M after the three-point bending test and the compressive side of specimen are shown in Figure 19. The predominant failure mode is layer buckling without fracture at the tensile side of testing.

## 4. Conclusions

This study contributes to the field of additive manufacturing by integrating continuous aramid fibers into PLA composites using a nozzle impregnation method. The results obtained within the scope of the study are listed below.

The voids and gaps between adjacent lines and layers decreased with decreasing layer height and hatch spacing. Similarly, the reinforcement fiber volume fraction in the composites increased by a closer packing of fibers at a decreased layer height and hatch spacing. It was concluded that the matrix penetration into the reinforcement fiber was limited, and the matrix material could not reach the center of the fibers.

The combination of a reduced layer height and narrower hatch spacing yielded the best mechanical performance, with a tensile strength of 410.25 MPa achieved at a 0.6 mm layer height and a 0.4 mm hatch spacing. This combination minimizes void formation, enhances fiber alignment, and strengthens interlayer adhesion, leading to superior mechanical properties.

The FTIR and XPS results show that atmospheric plasma modification can enhance the interfacial bonding strength by improving the surface morphology and increasing the content of polar groups on the fiber surface.

By combining optimized manufacturing conditions with atmospheric plasma treatment, the mechanical performance of continuous-aramid-fiber-reinforced PLA composites was enhanced.

## Figures and Tables

**Table 1:** 3D printing parameters of composites.

Sample Code	Hatch Spacing (mm)	Layer Height (mm)
1-08	1	0.8
1-06	1	0.6
1-04	1	0.4
08-08	0.8	0.8
08-06	0.8	0.6
08-04	0.8	0.4
06-08	0.6	0.8
06-06	0.6	0.6
06-04	0.6	0.4
06-06 m *	0.6	0.4

* 06-06 m refers to atmospheric-plasma-modified fiber.

**Table 2:** Fiber volume fraction of composites.

Sample Code	Fiber Content (vol%)
1-08	11.297
1-06	18.150
1-04	21.833
08-08	16.395
08-06	19.266
08-04	23.371
06-08	17.884
06-06	20.165
06-04	26.851

**Table 3:** XPS surface analyses.

Sample Code	XPS				
	C_1S_	N_1S_	O_1S_	O_1S_/C_1S_	N_1S_/C_1S_
Aramid—non-modified	78.7	1.3	20.0	0.25	0.016
Aramid—modified	77.4	1.4	21.2	0.27	0.019

**Table 4:** Distribution of functional groups (%).

Sample Code	XPS			
	C–C/C–H	C–O/C–O–C	O–C=O	Polar/Nonpolar
Aramid—non-modified	75.0	19.2	5.8	0.33
Aramid—modified	70.8	23.0	6.2	0.41

## Data Availability

The original contributions presented in this study are included in the article. Further inquiries can be directed to the corresponding author.
